# Syngeneic leukemia models using lentiviral transgenics

**DOI:** 10.1038/s41419-021-03477-2

**Published:** 2021-02-18

**Authors:** Nurit Keinan, Ye’ela Scharff, Oron Goldstein, Michael Chamo, Stefan Ilic, Roi Gazit

**Affiliations:** The Shraga Segal Department for Microbiology, Immunology, and Genetics, Faculty of Health Sciences; National Institute for Biotechnology in the Negev, the Ben-Gurion University of the Negev, Beer-Sheva, POB 84105 Israel

**Keywords:** Cancer models, Haematopoietic stem cells, Leukaemia

## Abstract

Animal models are necessary to study cancer and develop treatments. After decades of intensive research, effective treatments are available for only a few types of leukemia, while others are currently incurable. Our goal was to generate novel leukemia models in immunocompetent mice. We had achieved abilities for overexpression of multiple driving oncogenes simultaneously in normal primary cells, which can be transplanted and followed in vivo. Our experiments demonstrated the induction of primary malignant growth. Leukemia lines that model various types of leukemia, such as acute myeloid leukemia (AML) or chronic lymphocytic leukemia (CLL), were passaged robustly in congenic wild-type immunocompetent mice. These novel leukemia lines, which may complement previous models, offer the flexibility to generate tailored models of defined oncogenes of interest. The characterization of our leukemia models in immunocompetent animals can uncover the mechanisms of malignancy progression and offer a unique opportunity to stringently test anti-cancer chemotherapies.

## Introduction

Leukemias are a major blood cancer, and the most common cancer in children^1^. Leukemic cells originate from perturbed hematopoietic cells, and maintain their similarities to Hematopoietic Stem and Progenitor Cells (HSPCs)^2–5^. Understanding the basic biology of cancer and developing new treatments requires clinical and experimental progress in understanding the initiation, progression, and relapse of leukemia^6–10^.

Normal hematopoiesis follows a well-organized hierarchy^11^ through the differentiation of progenitors, which researchers formerly considered discrete sequential populations but now conceptualize them as a continuum landscape forming populations^12^. In this massive and complex process, which produces one million cells/s in humans cells may acquire genetic and epigenetic abnormalities that enhance proliferation or block differentiation. This leads to an excess of immature progenitors in the bone marrow (BM) and peripheral blood, thus causing different leukemic diseases, with often severe morbidity and mortality rates^13^. A variety of driving mutations and chromosomal aberrations lead to a range of leukemia types having correspondingly different pathophysiologies^14,15^. This diversity is due to differences in the driving oncogenes, in the cells-of-origin, and in the progression of each leukemia subtype.

There are four main categories of leukemias: chronic lymphoblastic leukemia (CLL), chronic myelogenous leukemia (CML), acute lymphoblastic leukemia (ALL), and acute myelogenous leukemia (AML). Each category may have clinically significant subtypes. Decades of intensive research has led to the development of advanced treatment options, including conventional chemotherapies and targeted treatments, which provide a cure for most types of ALLs in children^16^ and prolonged remission for CML^17^, but not for CLL or AML. Hence, an urgent need to better understand the biological basis of leukemia remains, both to prevent it and to develop new treatments. The experimental validation of new treatments begins with studying them in animal or cell models^13^.

When establishing a leukemia mouse model, one should note that leukemia subtypes correlate strongly with their driving oncogenes and with their cell of origin. Major oncogenic-translocations are present not only in mature cancer cells but also in the primitive stem cells and even normal HSCs of affected patients^18^. Therefore, the cell type from which the leukemia originates may have significant implications, as it is possible that different initiating cells can produce leukemias that appear similar yet respond differently to treatments. Interestingly, both the driving oncogenes and the cell-of-origin may determine the type of leukemia. For example, committed granulocyte-macrophage progenitors transformed by MLL-AF9 sustain much of their progenitor’s identity beyond the activation of a few stem-cell genes^19^. Studies of transgenic mice demonstrated that inducible overexpression of BCR/ABL cause specific B-cell leukemia when expressed in pre-B cells, but a totally different megakaryocytic disease when induced in more primitive cells^20,21^.

Mouse models have led leukemia research and drug development for over 70 years, and remain the most advanced tools and techniques even today^13^. The ability to isolate defined stem or progenitor populations, infect them in vitro, and transplant them into syngeneic mice enabled the rapid generation of numerous molecularly defined leukemia lines. Such transplant models were successfully produced with retroviral vectors^13,22^, yet these included active LTR enhancers that randomly activated additional genes. By contrast, lentiviruses (LV) offer an efficient delivery system and greatly reduced side effects thanks to self-inactivating LTR^23^.

In this study we used LVs to generate leukemia models in immunocompetent mice. Transducing an oncogenic mix simultaneously allowed various combinations of driving factors to be formed in the cells, as well as self-selection of the malignant ones, which produces a growth advantage in vivo. The emerging leukemia lines represented models to be characterized, studied mechanistically, and tested with leukemia treatments using a systematic and defined platform. Using this method, we established AML-like and a CLL-like leukemia-lines. Our results indicate that overexpression of multiple oncogenes in immune-competent mice can generate novel, “tailored” leukemia models. The ability to create different sub-types of leukemia models depends on the oncogenic factors transduced to the primary cells, thus providing a rapid way to generate a variety of molecularly defined leukemia lines.

## Results

**Fig. 1:**
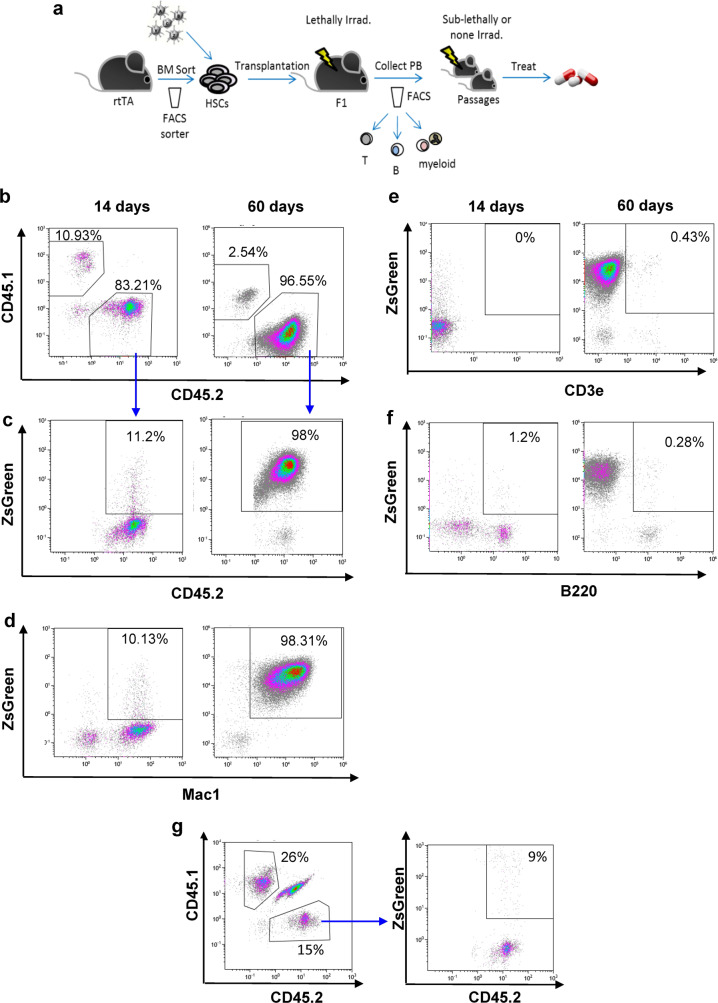
Overexpression of various oncogenes in HSPCs induces malignant growth in vivo. **a** Schematic representation of the experimental procedure. BM of CD45.2 rtTA mice sorted to obtain HSPCs. Candidate oncogenes were introduced in batches. Cells were transplanted into congenic mouse. FACS analysis of peripheral blood (PB) revealed the appearance and progression of leukemia. Passage into secondary and tertiary recipients established these models. **b**–**f** Representative FACS profiles of cells from the PB of ML23. Increased fraction of CD45.2^+^ZsGreen^+^ (**b**, **c**). Increase in Mac1^+^ (**d**), not in CD3e or B220 or **e**, **f** Same donor cells transduced with control-LV shown to yield no malignant growth (**g**). Data shown from one out of at least three independent experiments.

The overexpression of oncogenes can induce malignant growth in vivo. To produce a leukemia model in mice, we sorted hematopoietic stem and progenitor cells, infected them with a batch of LVs to induce the overexpression of multiple oncogenes, and transplanted them into syngeneic hosts (Fig. 1a).

Peripheral-blood (PB) analysis detected the progression of leukemia in terms of increased numbers of fluorescently labeled leukocytes. We characterized the leukemia type using major lineage markers, including Mac1, B220, and CD3e, to delineate myeloid, B-cells and T-cells, respectively (Fig. 1d–f). Importantly, transplantation of malignant cells into secondary and tertiary recipients established novel leukemia lines that enable the testing of in vivo treatments.

Figure 1b–f shows the generation of malignancy in immunocompetent mice following the overexpression of various oncogenes in HSPCs using our method. HSPCs were transduced with a mix of nine LVs, each of which inserted one of the following oncogenes: *Evi1*, *Glis2*, *HoxB5*, *HoxA9*, *Hlf*, *Meis1*, *MyCN*, *Prdm5*, and *Runx1*. These nine oncogenes were selected on the basis of combined data from previous studies that identified them as leukemia-driving oncogenes together with experimental data previously obtained by our group that identified these oncogenes among HSC transcriptional factors^24–29^. Primary recipients were lethally irradiated (X-Ray 850 RAD) and received fresh whole BM cells (for myelo-protection) together with the ex-vivo manipulated HSPCs (which had been rendered oncogenic by LV-mix transduction). The development and progression of leukemia were followed in the cell transplant recipients by fluorescence-activated cell sorting (FACS) analysis of regularly collected PB. Figure 1b is a representative FACS profile of ML23 showing strong chimerism of the donor cells (from a CD45.2 mouse) in the recipient mouse (being a heterozygotic F1 CD45.1 + CD45.2 congener). Increase of ZsGreen^+^ frequency within the CD45.2 population of the donor cells over time is demonstrated (Fig. 1c). Macrophage antigen complex-1 (Mac1) expression, with little or no B220 or CD3, indicated the myeloid linage of these cells (Fig. 1d–f). By contrast, when HSPCs were infected with control LV that expressed the ZsGreen gene but did not include oncogenes, CD45.2 donor cell levels remained low, no leukemia was observed, and the population of ZsGreen^+^ CD45.2 cells did not increase over time (Fig. 1g), suggesting that malignant growth indeed developed because of overexpression of the oncogenes and not because of the viral integration. PB was further examined two and three months after transplantation and similar results were obtained (data not shown).

### Primary malignant cells can passage leukemia lines

**Fig. 2:**
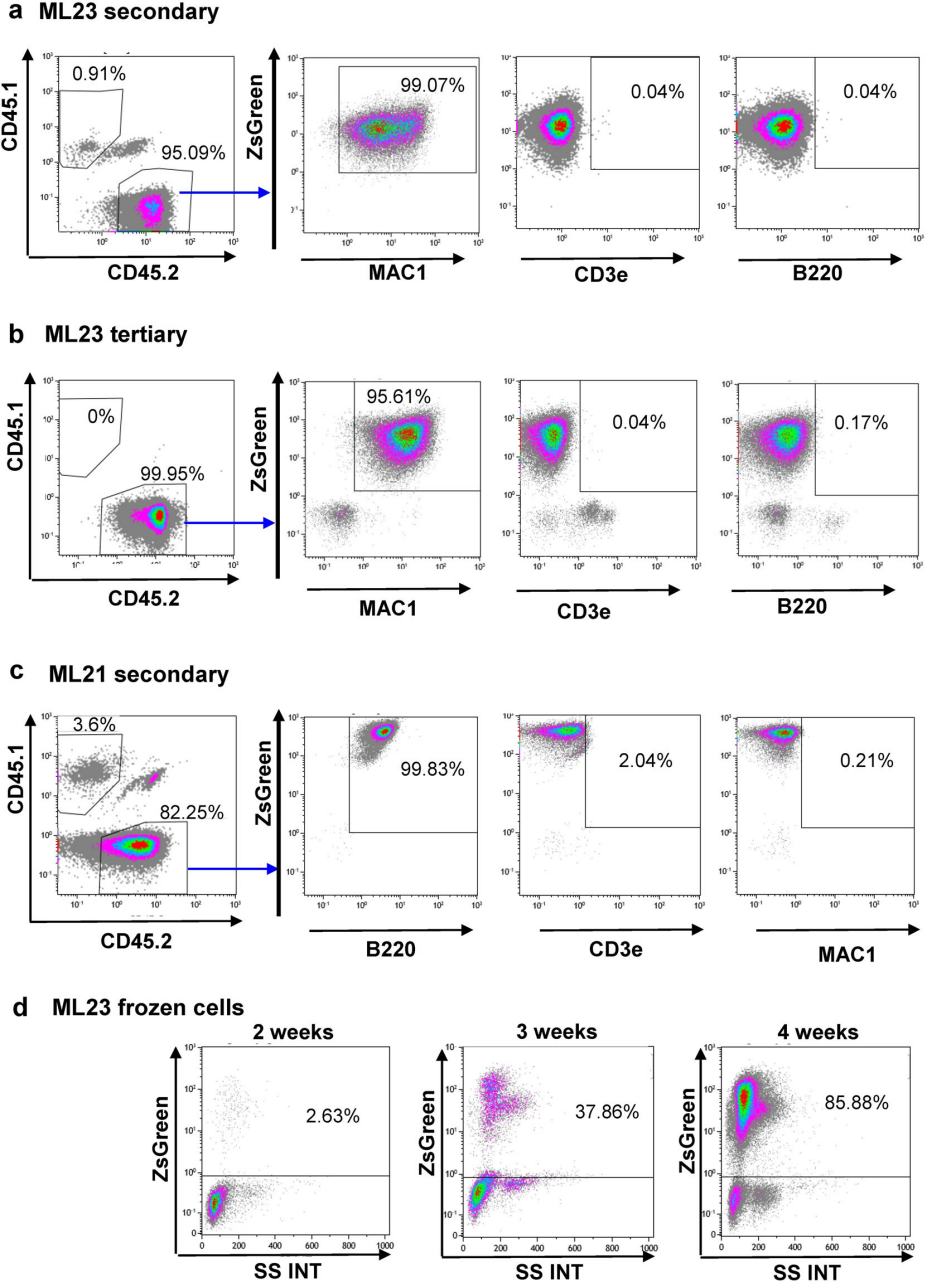
Leukemia lines can passage into WT mice. Representative PB FACS taken from secondary recipient 30 days after passage of ML23 (**a**), tertiary ML23 recipient (**b**), or ML21 (**c**). One million fresh cells were used for these passages. Plots show frequencies of the transplanted CD45.2 cells, expression of ZsGreen reporter, Mac1, CD3e, and B220 staining. Data shown from one out of at least three independent experiments. **d** Representative PB FACS of recipient mice after transplantation with previously frozen ML23 cells. Data shown from one out of at least three independent experiments.

The primary malignancy, as interesting as it is, does not provide a robust approach for additional experiments, as each primary recipient is unique. Passage of primary malignant cells from primary recipients into secondary hosts can establish a cohort of animals. In the current research, we considered the transfer of a million cells from primary mouse BM and their successful establishment in secondary hosts as a robust measure of passage-ability. Figure 2 shows the successful passage-ability of cells from the ML23 cell line into secondary (Fig. 2a) and tertiary (Fig. 2b) recipients. Strong chimerism of the donor cells (CD45.2) in the recipient mice (CD45.1 + CD45.2) is shown in the left panels. High ZsGreen expression in live CD45.2 donor cells is also shown in the middle panels; moreover, in the right panels, high CD45.2-Mac1 staining is demonstrated, indicating the stability of this myeloid leukemia. Figure 2c demonstrates another example involving a different leukemia line, namely, ML21. Here, secondary passage showed high CD45.2 levels, high ZsGreen expression, and high B220 staining, suggesting the B-cell leukemia sub-type (Fig. 2c).

Both ML23 myeloid leukemia and ML21 B-cell leukemia achieved robust growth in secondary and tertiary recipients, yielding millions of ZsGreen-tagged cells in BM and billions of such cells in spleens. These cells were aliquoted and stored frozen. The potency of frozen cells to transplant the disease further into other cohorts of mice was tested and found positive (Fig. 2d). This suggests that freezing prior to transplantation is a convenient option for expanding primary malignant cells and establishing substantial cohorts having similar leukemic growth for functional experiments. The robust ability to passage an established leukemia line is demonstrated in Supplementary Fig. 1. We tried to culture both ML21 and ML23 leukemia lines, but they did not grow for more than few days in vitro (data not shown).

### Identification of oncogene(s) in leukemic cells

**Fig. 3:**
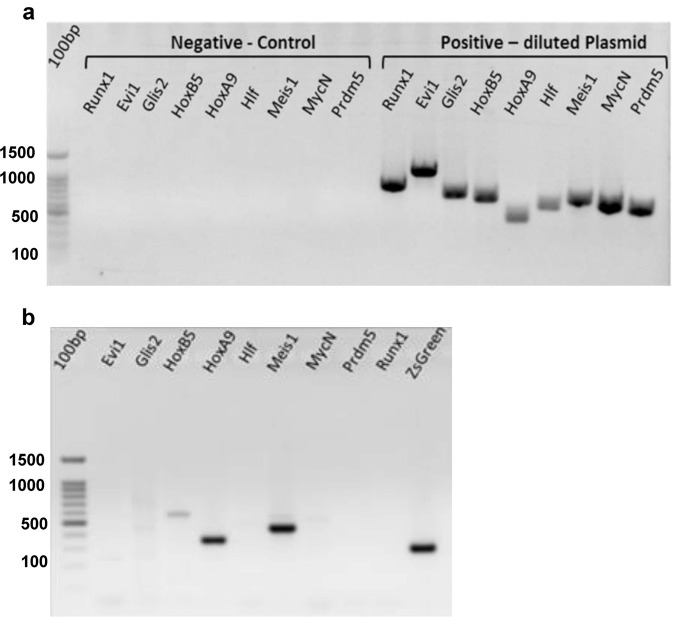
Using polymerase chain reaction (PCR) analysis to identify the driving oncogenes in the leukemic lines. **a** Control PCR w/o leukemia-line DNA, or diluted plasmids (1 ng). **b** PCR of ML23 detecting *HoxB5*, *HoxA9*, and *Meis1* oncogenes. The 100-bp ladder is on the left lanes. Data are from one of at least three independent experiments (*n* = 3).

Established leukemia lines may be derived from a few or many of the oncogenes used. Retrospective identification of the leukemia-inducing oncogenes was performed by polymerase chain reaction (PCR) analysis of genomic DNA, as the LVs were stably integrated. We purified genomic DNA from the leukemia line and performed PCR with specific primers for the oncogenes that were transduced into the donor cells (Fig. 3a, b). Controls without leukemia-line DNA (negative control) and with diluted plasmids (positive control Fig. 3a) were performed in parallel.

Representative PCR gel results revealed three specific bands corresponding to *HoxB5, HoxA9*, and *Meis1*, indicating that these driving oncogenes were gnomically integrated into the leukemia cells and suggesting their involvement in leukemia generated in ML23 mice. As expected, the PCR also revealed a positive band corresponding to ZsGreen in the ML23 line (Fig. 3b).

Importantly, this straightforward approach to identify driving factors can be used for any mix of LVs, and we have used it before in the reprogramming-experiments^30^. However, it should be noted that the presence of multiple oncogenes together with challenging PCR analysis of total genomic DNA required fine calibration of primers and reactions. Supplementary Figure 2 shows identification of integrated LVs in ML21, with some clear products and other possible non-specific bands.

### Characterization of the leukemia lines in vivo

**Fig. 4:**
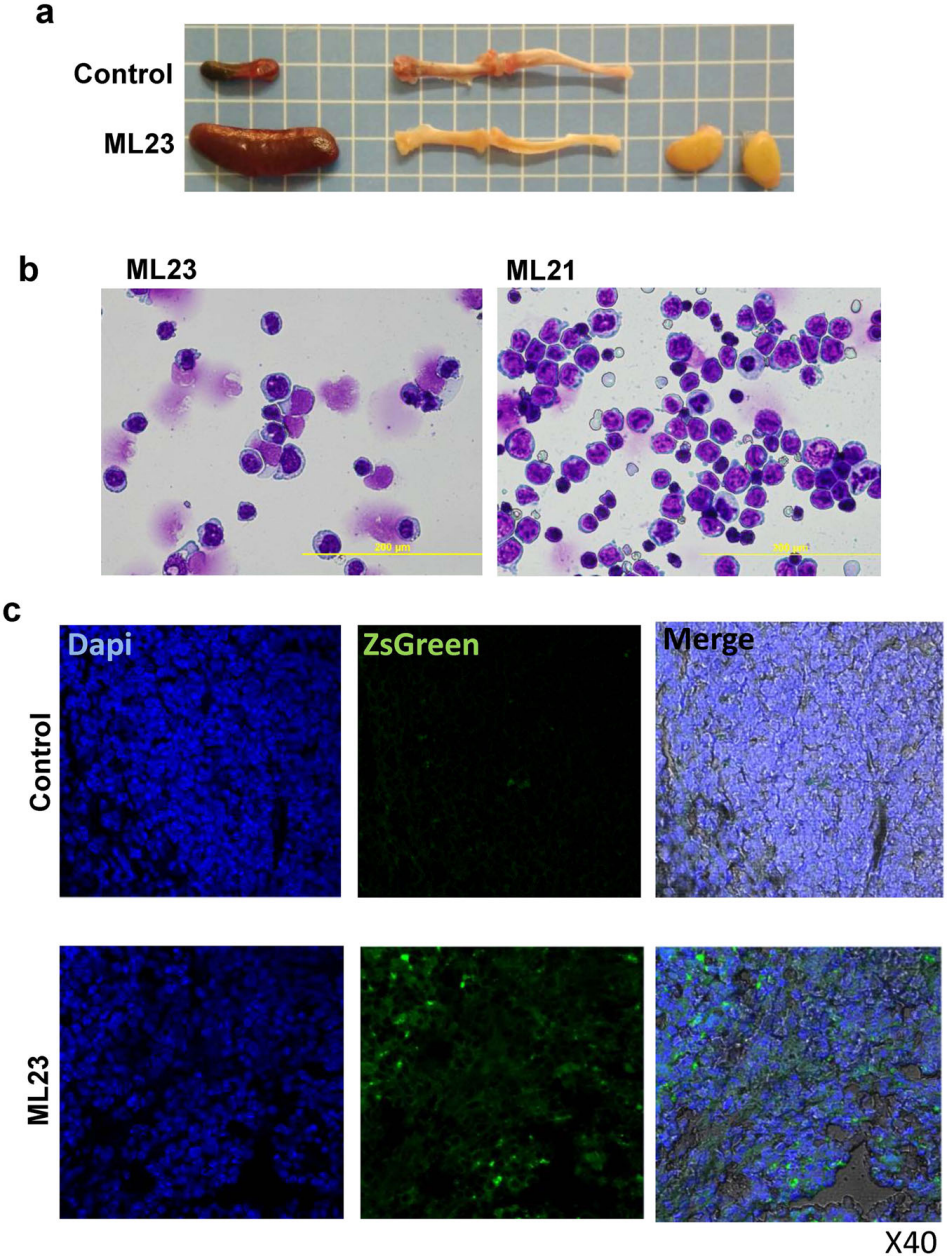
Leukemia pathophysiology and microscopic examination. **a** Enlarged spleen and lymph-nodes of an ML23 mouse, alongside bones lacking the normal reddish-hematopoiesis marrow are shown, normal spleen and bone marrow of a control-healthy mouse are presented for comparison (representative of *n* = 7 mice). **b** Giemsa-stained ML23 or ML21 cells at ×20–40 magnification. **c** Spleens sections from WT, or ML23 mice, with nuclear-staining with Dapi. Green fluorescence is detected from ZsGreen-tagged spleen cells in the ML23 mice only.

To further establish and characterize the new leukemia mice model, we conducted pathophysiological, histological, and microscopical examinations of the mice (Fig. 4). ML23 mice had an enlarged spleen, enlarged lymph nodes, and bones lacking the normal reddish-hematopoiesis marrow (Fig. 4a). Moreover, Giemsa staining, performed to examine the morphology of ML21 and ML23 leukemic spleen cells (Fig. 4b), revealed leukemic blasts-like cells. By analysis of ZsGreen expression in oncogenetic tissues taken from the leukemia lines, a bright labeling in the leukemic tissues was found (Fig. 4c), thus offering a convenient way to detect LV-infected cells in vivo.

### Leukemia lines respond to treatment

**Fig. 5:**
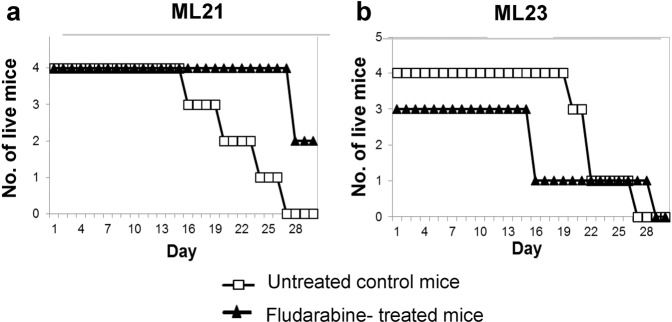
Response of leukemia models to conventional chemotherapy. Four-week survival curves for untreated control mice, and chemo-treated mice (Fludarabine 25 mg/kg). **a** ML21 mice with lymphoid-leukemia; **b** ML23 mice with myeloid-leukemia. Per experiment *n* = 7–8 mice per treatment. Data shown are from one of three independent experiments.

To test whether the novel models respond to conventional therapy, we examined their response to fludarabine, which is a common chemotherapy for CLL^31^. Cohorts of mice were transplanted with the established leukemia lines ML23 (myeloid-leukemia) and ML21 (lymphoid-leukemia). Since all mice were positively validated for malignant growth by FACS analysis of PB (not shown), they were grouped simply by their cages for control-groups or for Fludarabine-treatment. Treatment increased the survival of mice with ML21 lymphoid leukemia, as compared with controls (Fig. 5a). However, treatment had no statistically significant effect on mice with ML23 myeloid leukemia (Fig. 5b). Thus, the response of the ML21 model to fludarabine chemotherapy provides an early indication how these cell lines may aid in the identification of the therapies to which the models (and patients with similar pathologies) can respond. The ability of such leukemia lines to propagate and passage to cohorts of mice suggests the opportunity to examine new drugs for multiple types and sub-types of leukemia.

### Subpopulation with distinct stem-cell markers found in leukemic cells

**Fig. 6:**
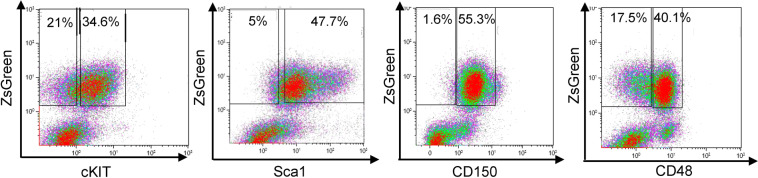
Leukemia cells contain a subpopulation with distinct stem-cell markers. Representative FACS plots of ML23 expression of stem cell markers cKit, Sca1, CD150, and CD48, showing subpopulations of cKit^−/+^Sca1^+^CD150^+^CD48^−/+^. *n* = 10 mice; Data are from one of at least three independent experiments.

Human leukemia is known to consist of a heterogeneous population of cells, especially in AML, where heterogeneity is common and functionally relevant. Leukemic ZsGreen-tagged cells from ML23 were examined by FACS to determine whether they manifested any heterogeneity and whether there was a subpopulation that resembles stem cells. Stem cell markers, such as cKit, Sca1, CD150, and CD48, were used to reveal any similarities between leukemic cells and HSCs. FACS profiles indicated heterogeneity with respect to the expression of cKit and CD48 surface markers, whereas the cells were usually Sca1^+^ (with some variation among cells) and uniformly CD150^+^ (Fig. 6). These findings demonstrate that, similarly to human myeloid leukemia, the ML23 line presents phenotypic heterogeneity that may correlate with stem cell-like appearance. Usage of additional surface markers will allow further resolution of the immune phenotype and possible functional heterogeneity among leukemic cells in this novel syngeneic mouse model.

## Discussion

### Generating novel models of leukemia by overexpression of specific oncogenes in immunocompetent mice

Generating mouse models for studying leukemia is important for basic research and for translational studies. Leukemia mouse models help unravel the in vivo consequences of activating multiple oncogenes in defined stem- or progenitor-cell populations. Leukemia lines are used in the pre-clinical testing of novel therapies. The success of current research in creating a method to generate novel leukemia models in immunocompetent mice opens the door to optimizing pre-clinical testing to target sub-types of the disease. These models benefit from a true in vivo microenvironment with high dependence on it. The essential leukemia- microenvironment interaction for leukemic cells survival and disease progression was demonstrated in previous studies^32–34^. The method described in this study is also applicable for generating leukemia models in other mouse strains and in other animals of interest. Importantly, this approach enables batch transduction of multiple oncogenes and the identification of those that most potently drive normal cells into malignancy in vivo.

**Fig. 7:**
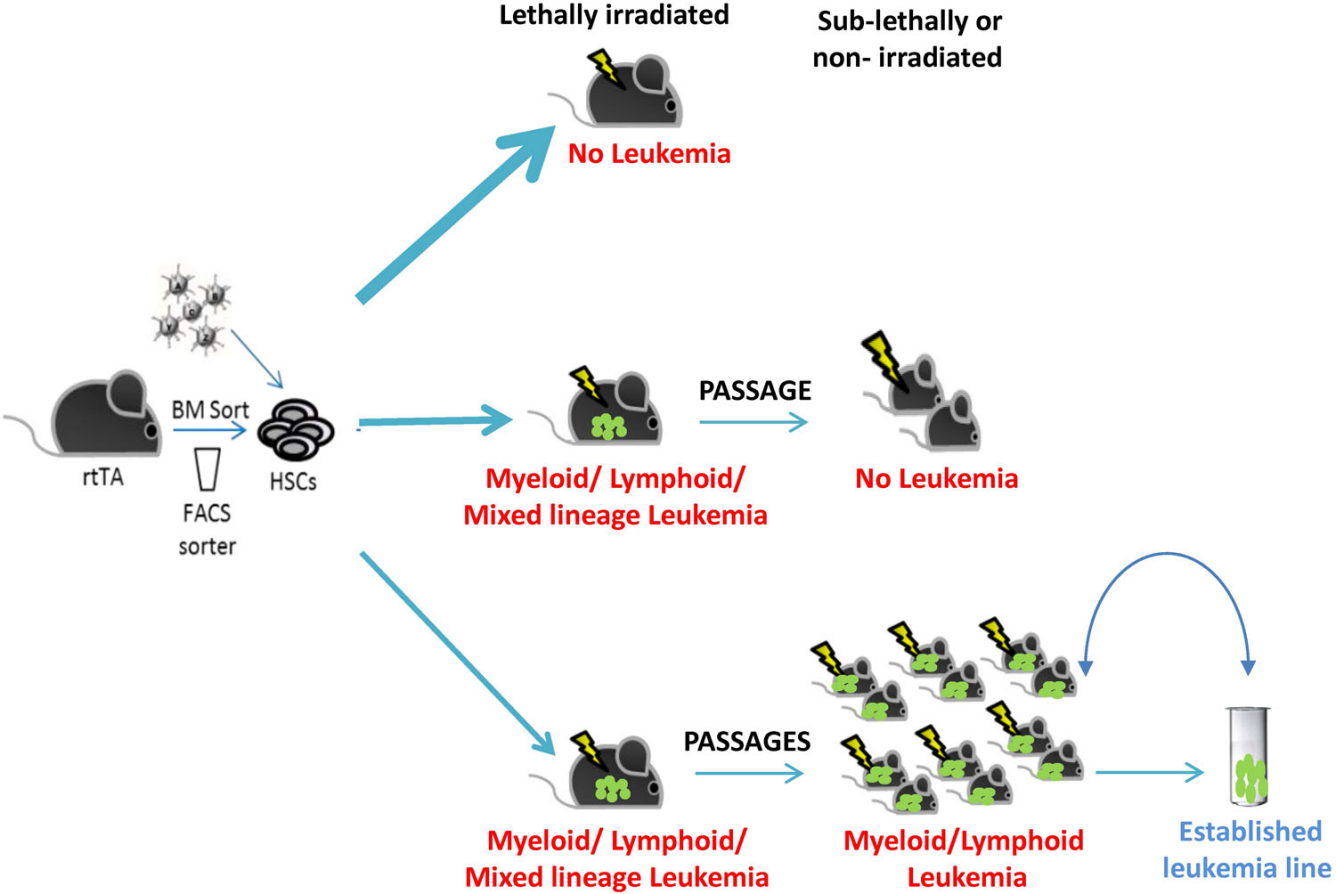
Generation of a multiple leukemia line in vivo. Schematic model demonstrating that overexpression of a mix of oncogenes in immunocompetent mice can induce malignant leukemia. Upper arrow: no leukemia develops. Middle arrow: initial growth suspected but no further passage. Lower arrow: leukemia is generated and passaged. Established leukemia-lines can easily transfer such models.

Following our experimental results, we suggest a platform for the generation of multiple leukemia-lines in vivo (Fig. 7). Overexpression of an oncogene mix in immunocompetent mice can lead to three main outcomes. First, continuous normal hematopoiesis may continue as a result of insufficient insertion of oncogene combinations into the genome. For example, in the case of only one oncogene achieving expression, tumor formation is rare and normal hematopoiesis may proceed despite minor perturbations. Second, leukemia may be generated in the primary mouse without further passaging into secondary recipient mice. Lack of passage may be due to low numbers of leukemic cells or insufficient expression of potent oncogenes capable of taking over normal HSCs in the recipient mice. Alternatively, lack of passage may reflect that even in a syngeneic situation, leukemia development depends on the modified microenvironment of the primary host, which is not present in secondary recipients. Third, malignancy in the primary mice may show the ability to passage to recipient mice. Leukemia able to passage into sub-lethally conditioned recipients enables the establishment of a leukemia-line that can provide cohorts of mice with the same genetic leukemia variants. Frozen leukemia-line cells represent a most convenient method for sharing such models.

### Oncogenic factors can dictate the type and dynamic progression of malignancy

In this study, we used a LV-oncogenic mix, which developed two main sub-types of leukemic models, one AML-like and the other CLL-like. The *HoxB5*, *HoxA9*, and *Meis1* oncogenes were inserted into ML-23 leukemic cell line DNA, which suggests that they were involved in inducing AML-like leukemia in our model (Fig. 3). These findings are consistent with previous studies that demonstrated the involvement of *HoxB5* and *HoxA9* in ovarian cancer and it has similarly been reported that *Hoxa9* and *Meis1* cooperatively induce malignancy, and AML in particular^35–37^. Interestingly, the oncogene in ML23 are known to relate with myeloid leukemia, however, ML21 CLL-like oncogenic analysis revealed *Runx1*, *Evi1*, *Glis2*, and *Hoxa9* (Supplementary Fig. 6). Some studies had reported *Hoxa9*, *Evi1*, or *Runx1* in lymphoid neoplasia^38–41^, but the combination of these four oncogenes to induce CLL-like leuekmia is novel. Notably, the ZsGreen-tagged reporter indicates oncogene expression, but does not provide information regarding each oncogene; using multiple tags emitting in various colors may improve the detection of multiple oncogenes. If the reporter fluorescent protein can be fused with the oncogene itself, further information regarding the concentration and sub-cellular localization of the proteins may be obtained.

Our method suggests a convenient way to include additional oncogenes of particular interest, as well as miRNAs, lncRNAs, and any other factor that can be cargo for LVs, into animal models. Our focus was on overexpression and we did not use knockout or ablation in this study for simplicity. The robustness of the malignant models arises from their combinatorial formation, specifically: one may include various candidate-drivers into a population of cells and reveal the most potent combination(s) retrospectively. This approach not only saves time and efforts, but truly allows larger-scale examinations of multiple candidate factors in one competitive system, as we did for reprogramming^30^. We suggest screening for multiple co-factors that drive malignancy in addition to a basic oncogenic driver that, by itself, cannot induce leukemia in the mouse.

The option of having multiple leukemias in one host is of great interest. In addition, the development of multiple leukemias can be minimized either by titration or by passage through secondary and tertiary hosts where only the most robustly growing leukemia would prevail. However, in some cases, more than one leukemia may occur in one primary host, as also sometimes occurs in human patients, and this may be of interest for future studies. Interestingly, recent studies suggest that leukemia may include multiple clones evolving in parallel and having divergent mutations, not just a linear serial-accumulation of mutations^42,43^. Our method can be used to further study such parallel clonal evolution models as one may create multiple HSPCs with divergent oncogenes in the same mouse, mimicking the situation in the human disease.

### Leukemia models can reveal heterogeneity

Our findings demonstrate that AML-like ML23 line leukemic cells contain a subpopulation with distinct stem-cell markers. Whereas HSCs are Lin^−^ Sca1^+^cKit^+^CD48^−^CD150^+^ and MPP linage is Lin^−^ Sca1^+^cKit^+^CD48^+^CD150^−^, the ML23 leukemia cell linage is cKit^−/+^Sca1^+^CD150^+^CD48^−/+^, which suggests heterogeneous subpopulations with respect to the cKit and CD48 markers. The cKit protein (also known as CD117) is a receptor for stem-cell-factor (SCF), which is a major cytokine for stem- and progenitor-cells^44,45^. The CD48 protein (also known as SLAMF2, and BLAST-1) is a 2B4 ligand^46,47^. In this study, our oncogenic-transcription factor overexpression may directly regulate cKit or CD48 expression levels, which in turn can affect cell malignancy. The cKit protein may drive cell proliferation whereas CD48 may provide protection from cytotoxic immune cells^48^. Importantly, a sub-population that exhibits low expression of cKit is of particular interest, as previous studies demonstrated that HSC function is strongly affected by even small changes in cKit signaling^49,50^. Conversely, functional and transcriptome studies in HSCs demonstrated that low levels of cKit expression display enhanced self-renewal properties compared with high cKit expression^50^. Moreover, it has been reported that a poorer prognosis in multiple myeloma is correlated with a lack of cKit, but also with beta-2 microglobulin, translocation t (4; 14), and CD221 (IGF-1R)^51^. Another study reported a correlation between a poor prognosis and low or absent cKit and CD56 expression on malignant plasma cells in newly diagnosed multiple myeloma patients^52^. Hence, low levels of cKit may correlate with the ability to passage the leukemia line easily into recipient mice and with the severe disease phenotype, as the cells turn from SCF-dependent cKit-high into SCF-independent cKit-low during malignant progression.

The CD48 protein is known to be involved in immune cell activation^53,54^, with high binding affinity to natural killer (NK) cells activating receptor 2B4. Hence, CD48 expression levels could affect NK-mediated cytotoxicity. Indeed, it has been previously reported that CD48 down-regulated by PML-RARA and AML-ETO oncogenic fusion proteins provides leukemia cells with immune evasion properties^55^. Moreover, TGF-β stimulation in MEG-01 cells, which are derived from a CML patient having BCR-ABL fusion protein, and in U937 cells, which are derived from lymphoma, results in suppressed CD48 expression levels and lower susceptibility to NK targeting^48^. Nevertheless, 2B4 are special receptors, capable of activating mature peripheral NK-cells while having inhibitory functions in immature NK-cells that may be prevalent in the BM^56^. These findings suggest the involvement of CD48 expression level heterogeneity, as observed also in our study, in promoting leukemia by escaping from NK- mediated killing, thus demonstrating once more the significance of using immunocompetent mice.

## Conclusions

We demonstrated the ability to create a mouse model for the formation and development of leukemia through the transduction of multiple LV-oncogenic factors into primary cells. Cloning of oncogenic factors is relatively simple, and the limitation of an ~4 kb open reading frame insert in LVs^23^ is sufficient for most genes. It is also relatively easy to introduce various mutations using molecular biology. Thanks to syngeneic mice, one can passage the leukemic cells to immune-competent secondary and tertiary recipients and track leukemia propagation for months. Established leukemia-lines, such as ML21 and ML23, can be shared easily by shipment of frozen cells that can then be transplanted into any C57BL/6 mouse. Transgenic leukemia models can greatly enhance the testing of new treatments, especially with respect to possible multiple lines that may resemble the variabilities among patients, and because they enable researchers to work with immunocompetent animals in the cancer immunotherapy era. Tailored models can further enhance the understanding and treatment development for leukemia sub-types for which no efficient therapy is currently available.

## Materials and methods

### Cloning

All plasmids were constructed using PCR amplification. Conventional cloning was used to introduce oncogenes of interest after the tet-responsive promoter (TRE) and bearing a ZsGreen fluorescent reporter based on the pHAGE2 vector^57^. The restriction and ligation reactions were performed using NEB enzymes (Ipswich, MA). PrimeSTAR Max DNA Polymerase (Takara Bio, Saint-Germain-en-Laye, France) was used for PCR-based cloning.

### Lentiviruses

HEK293T cells were plated in 10 cm plates in Dulbecco’s modified eagle medium (DMEM) containing 10% serum, pen-strep, 1% l-glutamine, non-essential amino acids, and sodium pyruvate (all from Biological Industries, Beit Haemek, Israel). The following day, cells were transfected using 45 µL homemade polyethylenimine (PEI), 12 µg of pHAGE2 lentivector, and 3 µg of packaging plasmids Tat, Rev, Hgpm2, and Vsvg in a 1:1:1:2 ratio in 800 µl of DMEM without additives. The medium was replaced the following day and then collected and replaced daily for another 3 days. The collected medium was centrifuged for 5 min at 2000 revolutions per min (rpm) and filtered through a 0.45-µm Thin-wall, Ultra-Clear filter. The supernatant was then transferred into 25 × 89 mm ultracentrifuge tubes (Beckman Coulter, CA) and centrifuged at 4 °C for 90 min at 17,000 rpm. Supernatant was carefully discarded and the residual volume was vortexed and incubated on ice for 1 h. Aliquots of 50 µl were stored at −80 °C. Control LV were produced containing only ZsGreen-gene, without oncogenes.

### Animals

Mice were kept in the institution’s specific-pathogen-free (SPF) unit, and all experiments were performed in accordance with Ben-Gurion University and Israel state Institutional Animal Care and Use Committees approval. Transgenic rtTA CD45.2 mice (Jax strain 6965, expressing m2-rtTA knocked in the Rosa26 locus on a C57BL/6 genetic background) were euthanized by isoflurane and spinal dislocation, their hips, femur, and tibia where removed, crushed by crater and pestle in 1 mL sample medium (SM: phosphate-buffered saline (PBS), 2% serum, 2 mM ethylenediaminetetraacetic acid (EDTA)), suspended in another 2 mL SM, and pipetted roughly. The supernatant was strained (70–100 µm mesh filter) to remove aggregates and larger debris and then centrifuged at 1600 RPM (500 g) for 5 min, and re-suspended in 5 ml SM at room temperature (RT). Using a plastic Pasteur pipette, 3 ml Histopaque^®^-1083 (Sigma-Aldrich, Israel) was loaded to the lower phase of the tube and centrifuged for 30 min at 450 g while deceleration/break were disabled. The distinct interphase containing mononuclear cells was transferred to a new tube and the histopaque residue was washed off. The supernatant was re-suspended in 200 µl SM, for staining. In some of the experiments, cKit^+^ cells were enriched using MidiMACS magnetic beads (Miltenyi Biotec, Germany), according to the manufacturer’s protocol.

### FACS

Antibodies used: CD45.1-Apc, CD45.2- PacBlue, Ter119-PerCPCy5.5, Mac1-PeCy7, B220-ApcCy7, CD3e-PE, cKit-ApcCy7, Sca1-APC, CD150-PeCy7, CD48--PerCPCy5.5, Gr1-PacBlue, and CD19-APC (all from Biolegend, San Diego, CA). Cells were stained on ice for 30 min, washed, filtered through a 70-µm filter, and analyzed using Fluorescence-Activated Cell Sort machine (FACS). Routine analysis was performed by using 4-laser Gallios (Beckman Coulter) and the Kaluza analysis software (Version 2.1). Sorting used of a 6-laser, BD FACSAria™ III sorter.

### Tissue culture

Primary HSPCs were grown in Biotarget medium (Biological Industries, Israel), supplemented with 1% l-glutamine, penicillin-streptomycin, sodium-pyruvate, non-essential amino acids (Biological Industries, Israel), β-mercaptoethanol (Sigma Aldrich, Israel), and 10 ng/ml SCF, TPO, IL3, and Flt3-L (Peprotech, Israel) cytokines. Polybrene (8 μg/ml) has added to enhance lentivirus infection. Primary sorted cells were plated (5000–15,000) in 96 U wells, following transfection by a 10–20-µl concentrated mix of either nine oncogene-containing LV or the control LV. Twenty-four hours later, doxycycline was added (1 µg/ml) to induce expression.

### Transplantation

Donor leukemic CD45.2 cells or control cells were transplanted into new hosts (chimeric F1 type mice presenting CD45.1 + CD45.2) accompanied by competitor CD45.1 cells. Cultured donor cells and WBM of competitor cells were mixed and intravenously (iv) injected into the tails of lethally irradiated host mice. BM or spleen cells were extracted from ML23 and ML21 mice, 1 million fresh cells were used to passage the leukemia line to secondary and tertiary recipient. For cryopreservation, BM or spleen cells from leukemic ML23 or ML21 mice were aliquted to 40 million cells per tube in NutriFreez D10 Cryopreservation medium (Biological Industries, Israel). The cells were kept frozen in −80 °C. Frozen cells were defrost and counted with trypen blue, 1 million viable cells were injected per mouse.

### Blood sampling

Mice were tail bled into a 150-µl Alsever’s solution. Samples where then treated with 10 ml Ammonium-Chloride-Potassium (ACK) Lysing Buffer for 2 min and centrifuged (1600 rpm) for 5 min. The supernatant was removed, washed in 5 ml SM, centrifuged again, and finally plated in a 96-U well for 1–3 h for staining. Blood panel staining: CD45.1-Apc, CD45.2-PacBlue, Mac1-PeCy7, B220-ApcCy7, and CD3e-PE (all from Biolegend, San Diego, CA).

### DNA extraction and PCR

BM was extracted as described, washed with SM, centrifuged at 2000 × *g* for 5 min, and re-suspended in SM. Genomic DNA was purified using a special kit (gSYNC DNA Extraction Kit obtained from Geneaid, Taiwan). The following forward (f) and reverse (r) primers were used to identify integrated factors: for *Glis2* integrated into ML21, f-tctgcacctacctgcttcct, r-gcttgacatgatggtcgttg; for *Meis1* integrated into ML21, f-ttcacttgaaggatggtaagtcc, r-tccactcgttcaggaggaac; and for *ZsGreen*, f-ccacgagtccaagttctacgg, r-gtcaggtgccacttctggttc. For each of the nine following oncogenes, the internal ribosome entry site (IRES) sequence (r-cacaccggccttattccaag) was used as the reverse primer and the forward primers were: *Runx1*, cctccttgaaccactccactg; *Evi1*, gaacccacacaggagagcaac; *Glis2*, ctaaaccaccactgcccactc (ML23); *Hoxb5*, agtccctgccctgcactaac; *HoxA9*, gccttctccgaaaacaatgc; *HLF*, atacaccgagtcccattgacc; *Meis1*, agaccagtccaaccgagcag (ML23); *MycN*, ccatccatcagcagcacaact; and *Prdm5*, tgggaccctgagagttcacatc.

The amplifications were performed at 94 °C for 5 min, followed by 35 cycles at 94 °C for 10 s, 60 °C for 25 s, and 72 °C for 1 min, followed by 72 °C for 1 min. PCR products were resolved on agarose-gel and visualized using ethidium bromide.

### Microscopy

Spleens were harvested from healthy control mice (6–10 weeks old) or leukemic mice (2–3 weeks post-transplantation). Organs were fixated in 4% paraformaldehyde (PFA) for 6 h at 4 °C. Following fixation, spleens were moved to a 30% sucrose solution. Tissues were coated with optimal cutting temperature (OCT) compound and stored in plastic chambers for cryostat use at −80 °C. Sections (20 µm thick) were made with a Cryostat (Leica). Tissues sections were stained with DAPI (4′,6-diamidino-2-phenylindole dihydrochloride; 1 μg/ml) for 1 h at RT. Slides were mounted with a cover-glass and images were taken with an Olympus Confocal Microscope using Olympus Fluoview FV1000.

### Fludarabine treatment

Fludarabine (25 mg/kg) was injected intraperitoneally (ip) three times into treated mice. Sterile PBS (200 µl) was ip injected three times into untreated control mice. Survival was inspected daily throughout the duration of the experiment. 7–8 mice were used per treatment, per experiment.

## Supplementary information

Supplementary file
