# Supplementary material for: Syngeneic leukemia models using lentiviral transgenics
Source: Cell Death Dis. 2021 Feb 18;12(2):193. doi: 10.1038/s41419-021-03477-2 (PMC7893004; doi:10.1038/s41419-021-03477-2)
Supplement: Supplementary file 1 — Supplementary file [file 41419_2021_3477_MOESM1_ESM.pdf]

Supplementary file of

Syngeneic leukemia models using lentiviral transgenics

Nurit Keinan[1], Yeela Scharff[1], Oron Goldstein[1], Michael Chamo[1], Stefan Ilic[1], Roi Gazit[1]

[1]The Shraga Segal Department for Microbiology, Immunology, and Genetics, Faculty of Health Sciences; National Institute for Biotechnology in the Negev; the Ben-Gurion University of the Negev, Israel POB 84105

Seven figures with captions, and one table

Supp 1

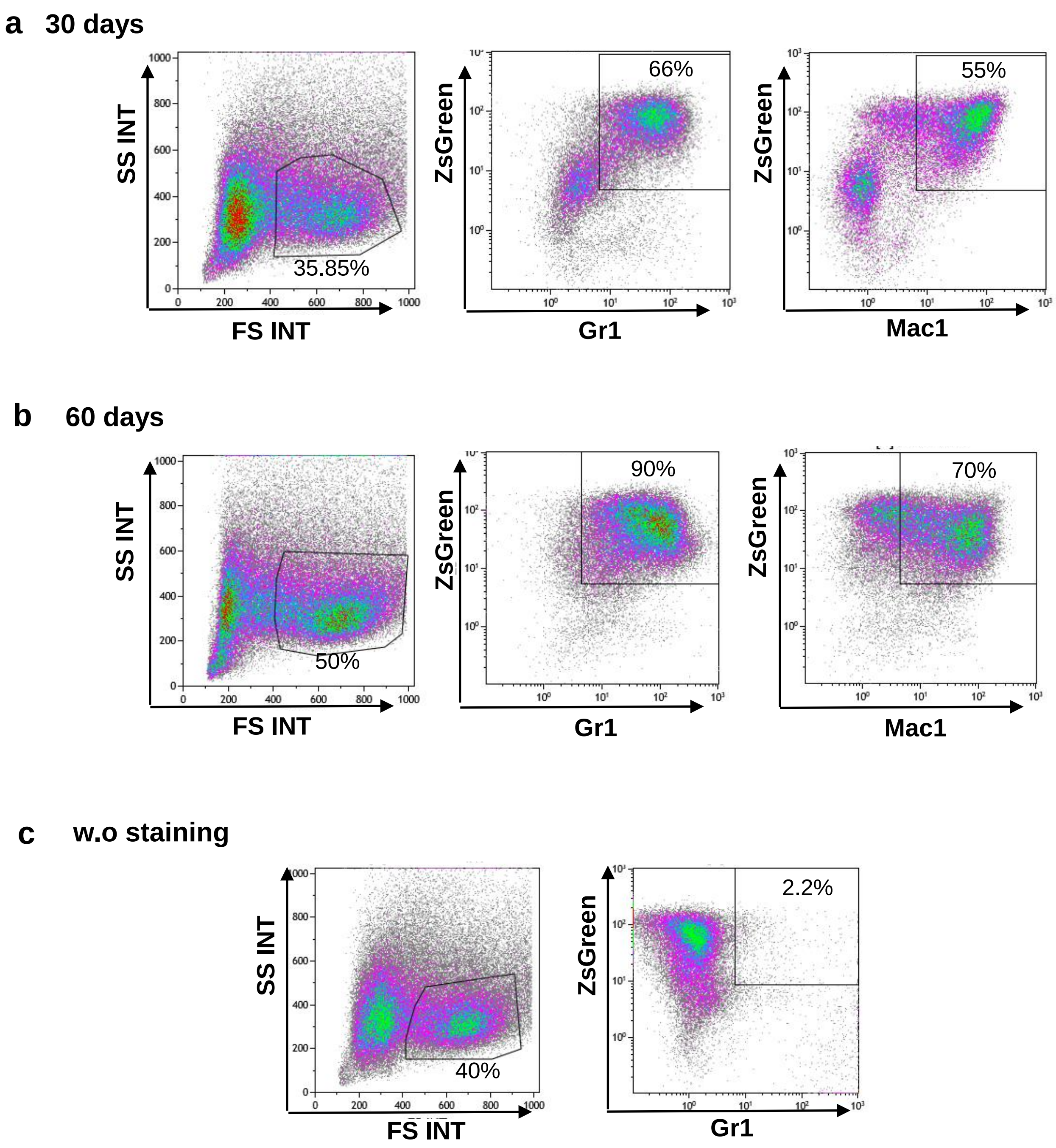

Supp 1. **ML23- bone marrow cells express MAC1 and GR1 myeloid markers.** Representative FACS plots of BM cells extracted from ML23 recipient's mouse showing (a) GR1 staining additional to MAC1 staining 30 days after transplantation and (b) an increased staining of both MAC1 and GR1 60 days after transplantation. (c) As a negative control the BM cells were analysed also w/o GR1 staining showing low GR1 expression as expected. n=12 mice; Data shown from one out of at least 3 independent experiments.

Supp 2

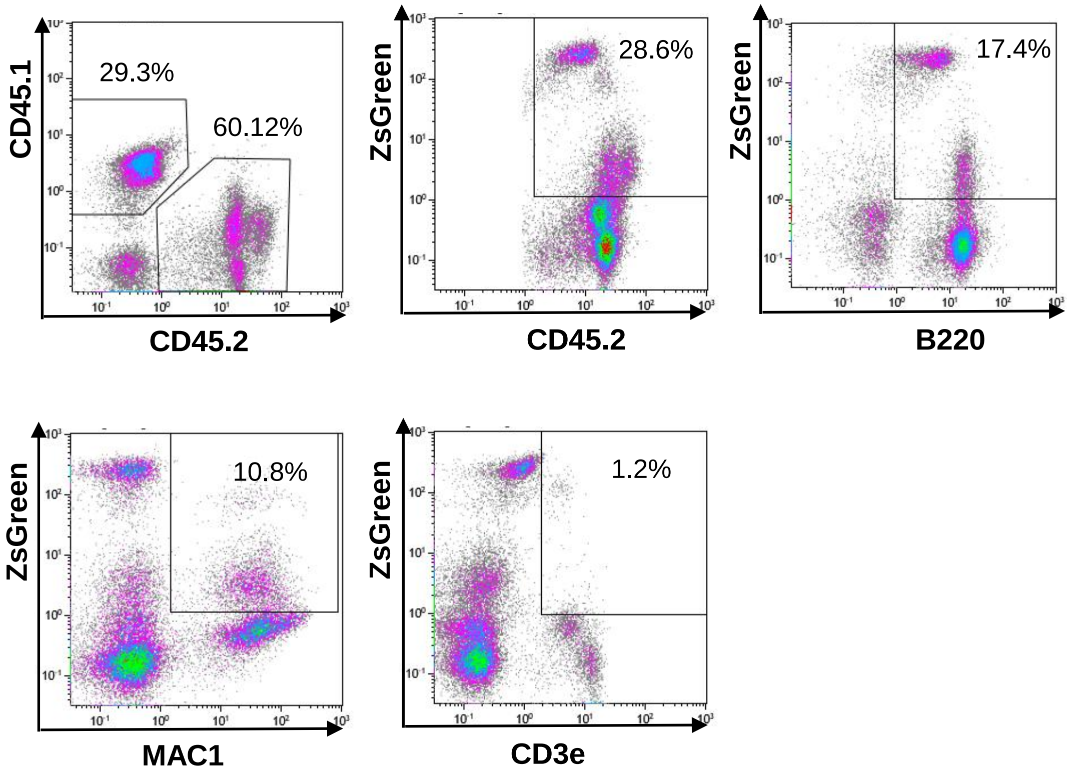

Supp 2. Mix lineage leukemia phenotype can be shown in the early stages of leukemia progression. Representative FACS plots of PB of mouse 21 (ML21) after transplantation of donor cells (45.2) over-expressed with “onci” lentivirus mix showing the following: donor cells (CD45.2) over-expressed with oncogenes is successfully transplanted into healthy mice, donor cells (CD45.2) show reporter gene (ZsGreen) expression, a moderate increase in both B22O and MAC1 staining and low CD3e staining. Among the 4 mice used in this initially experiment, 1 mouse developed myeloid leukemia (ML23), 1 mouse developed mix lineage leukemia at this early experimental stage (ML21), one mouse was found to be dead and the other mouse did not develop significant leukemia. Data shown from one out of at least 3 independent experiments.

Supp 3

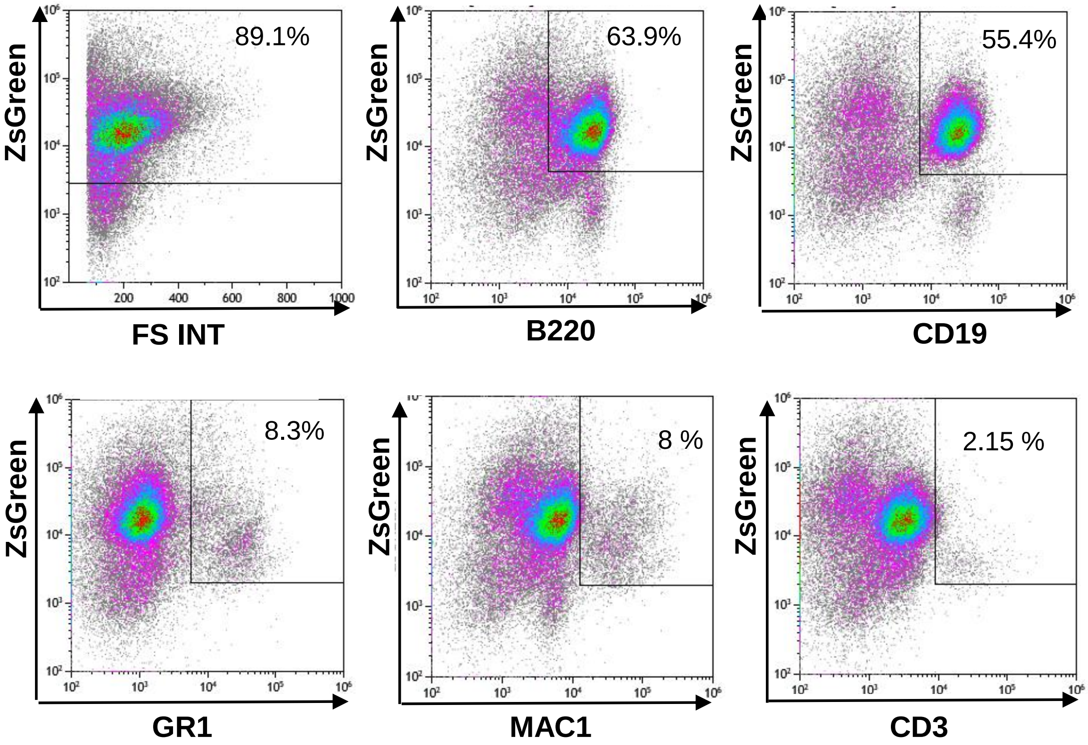

Supp 3. leukemia line 21 can passage into tertiary recipients.
Representative PB FACS plots of tertiary recipient mouse 30 days after BM transplantation, showing high expression of reporter gene (ZsGreen), high B220 and CD19 staining, mid-low GR1 and MAC1 staining and low CD3 staining. n=12 mice; Data shown from one out of at least 3 independent experiments.

Supp 4

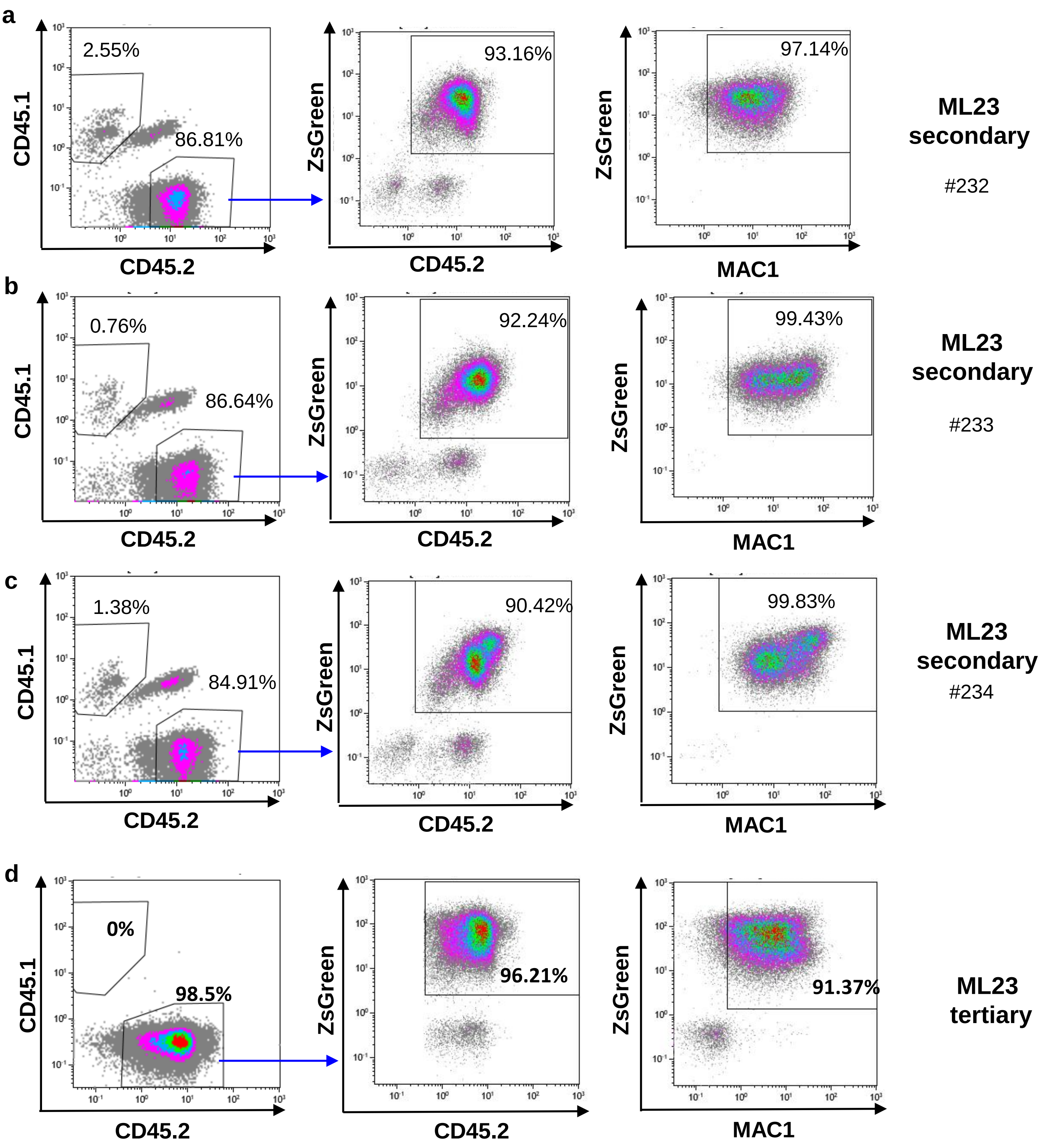

Supp 4. A robust leukemia can passage to all recipients' mice. (a, b and c) a, b, and c represent PB FACS plots of 3 secondary recipients' mice 30 days after being transplanted with ML23 BM and spleen cells. 15 out of 15 ML23 secondary recipient mice developed robust leukemia. High expression of transplant cells (CD45.2), high expression of reporter gene (ZsGreen) and high MAC1 staining is demonstrated, data shown from one out of at least 5 independent experiments. (d) Representative PB FACS plots of tertiary recipients mice 30 days after BM transplantation showing high expression of transplant cells (CD45.2), high expression of reporter gene (ZsGreen) and high MAC1 staining, 15 out of 15 ML23 tertiary recipient mice developed robust leukemia, data shown from one out of at least 5 independent experiments.

Supp 5

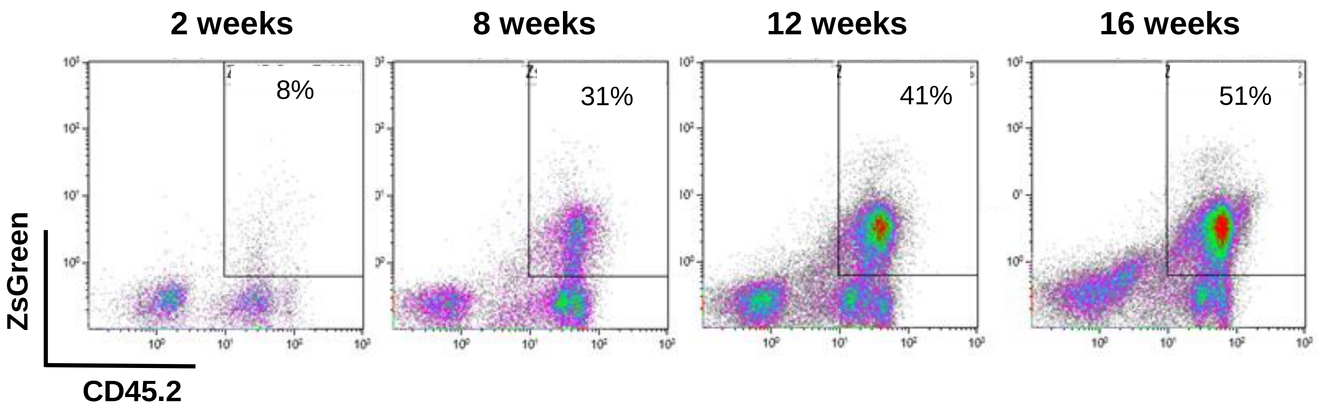

Supp 5. Overexpression of oncogenes in HSCs induces increase in malignant growth over time. Representative FACS plots of PB show increased expression of reporter gene (ZsGreen) over time after transplantation into healthy F1 (CD45.1+CD45.2) mice from donor cells (CD45.2) over-expressed with oncogenes. n=12 mice, data shown are from one of at least 5 independent experiments.

Supp 6

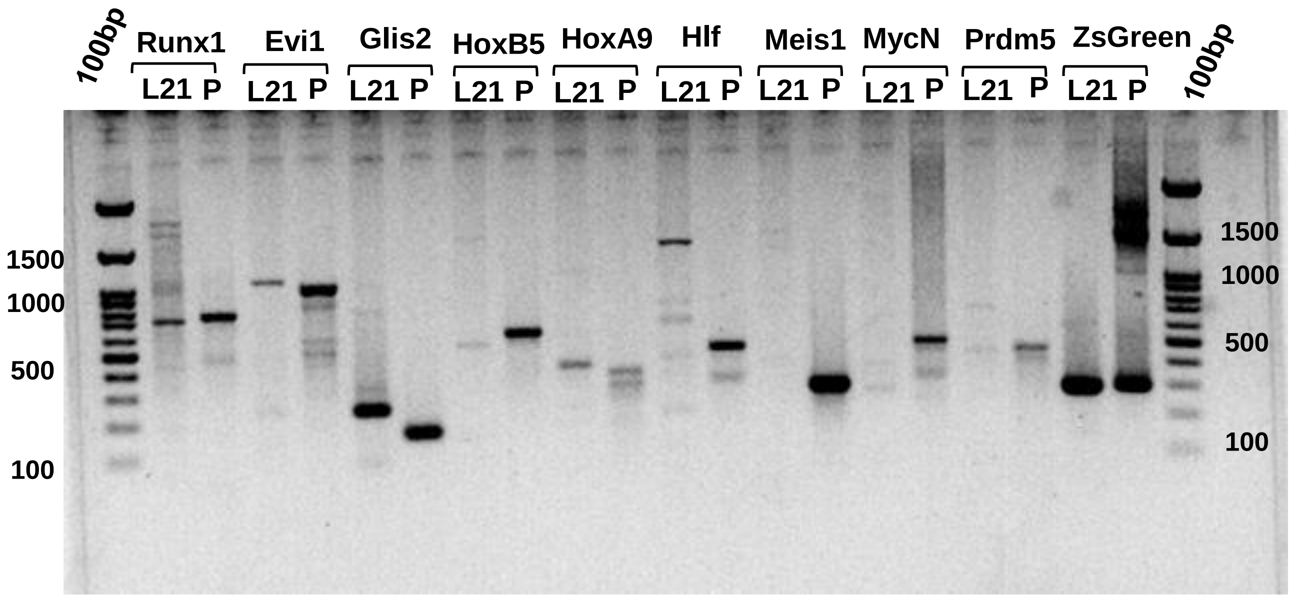

Supp 6. PCR enabled identification of driving oncogenes in ML21 leukemic lines. Unique sets of primers were designed for PCR analysis on ML21- bone marrow extraction (indicated as L21) or on diluted plasmids as positive control (indicated as P). Representative PCR results of ML21 mice showing Runx1, Evi1, Glis2 and Hoxa9 oncogenes are expressed and inserted within the leukemic cells genome out of the whole initially nine oncogene LVs mix infected the cells (n= 3). Glis2 which is not at the same size as the plasmid amplicon was also verified by Sanger sequencing. The last lane (ZsGreen) is a positive control of the quality of the sample.

Supp 7

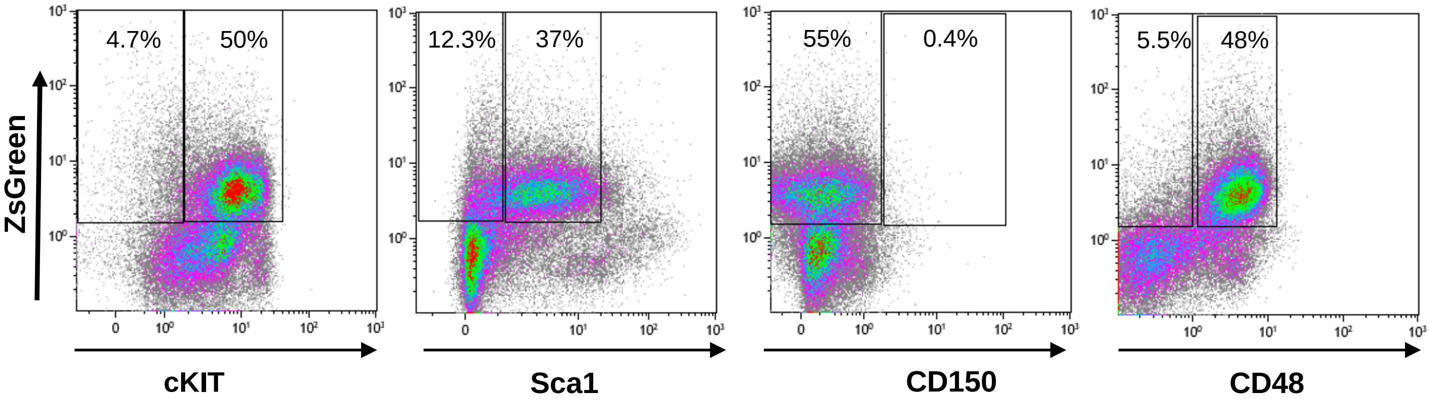

Supp 7. Leukemia cells contain a subpopulation with distinct stem-cell markers. Major stem cell markers profile (cKit, Sca1, CD150, CD48) were analyzed in the peripheral blood (PB) of ML21 mice. Representative fluorescence-activated cell sorting (FACS) showing a clear subpopulation that specifically demonstrates $cKit^{+}Sca1^{-/+}CD150^{-}CD48^{+}$. n= 3 mice; Data are from one of at least three independent experiments.

Supplementary Table 1: antibodies and cat#

Anti	Fluorophore	Clone	Lot	Supplier
c-Kit	APC-cy7	2B8	B231077	BioLegend
Sca1	APC	D7	B210898	BioLegend
CD150	PE-cy7	TC15-12F12.2	B210492	BioLegend
CD48	PC5.5	HM48-1	B229042	BioLegend
Lineage cocktial	Pacific-Blue		B223279	BioLegend
Mac1(CD11b)	PE-cy7	M1/70	B249268	BioLegend
B220	APC-cy7	RA3-6B2	B217170	BioLegend
CD3e	PE	145-2C11	C0031122[illegible]	Tonbo
Ter119	PC5.5	TER-119	B208036	BioLegend
CD45.1	APC	A20	C0453103[illegible]	Tonbo
CD45.2	Pacific-Blue	104	B169087	BioLegend
